# Carbohydrate Targets for CAR T Cells in Solid Childhood Cancers

**DOI:** 10.3389/fonc.2018.00513

**Published:** 2018-11-12

**Authors:** Claudia Rossig, Sareetha Kailayangiri, Silke Jamitzky, Bianca Altvater

**Affiliations:** ^1^Department of Pediatric Hematology and Oncology, University Children's Hospital Muenster, Muenster, Germany; ^2^Cells-in-Motion Cluster of Excellence (EXC 1003-CiM), University of Muenster, Muenster, Germany

**Keywords:** CAR T cells, childhood cancer, sarcomas, gangliosides, glycosylation

## Abstract

Application of the CAR targeting strategy in solid tumors is challenged by the need for adequate target antigens. As a consequence of their tissue origin, embryonal cancers can aberrantly express membrane-anchored gangliosides. These are carbohydrate molecules consisting of a glycosphingolipid linked to sialic acids residues. The best-known example is the abundant expression of ganglioside G_D2_ on the cell surface of neuroblastomas which derive from G_D2_-positive neuroectoderm. Gangliosides are involved in various cellular functions, including signal transduction, cell proliferation, differentiation, adhesion and cell death. In addition, transformation of human cells to cancer cells can be associated with distinct glycosylation profiles which provide advantages for tumor growth and dissemination and can serve as immune targets. Both gangliosides and aberrant glycosylation of proteins escape the direct molecular and proteomic screening strategies currently applied to identify further immune targets in cancers. Due to their highly restricted expression and their functional roles in the malignant behavior, they are attractive targets for immune engineering strategies. G_D2_-redirected CAR T cells have shown activity in clinical phase I/II trials in neuroblastoma and next-generation studies are ongoing. Further carbohydrate targets for CAR T cells in preclinical development are O-acetyl-G_D2_, NeuGc-GM3 (N-glycolyl GM3), G_D3_, SSEA-4, and oncofetal glycosylation variants. This review summarizes knowledge on the role and function of some membrane-expressed non-protein antigens, including gangliosides and abnormal protein glycosylation patterns, and discusses their potential to serve as a CAR targets in pediatric solid cancers.

## Introduction

**Figure 1:**
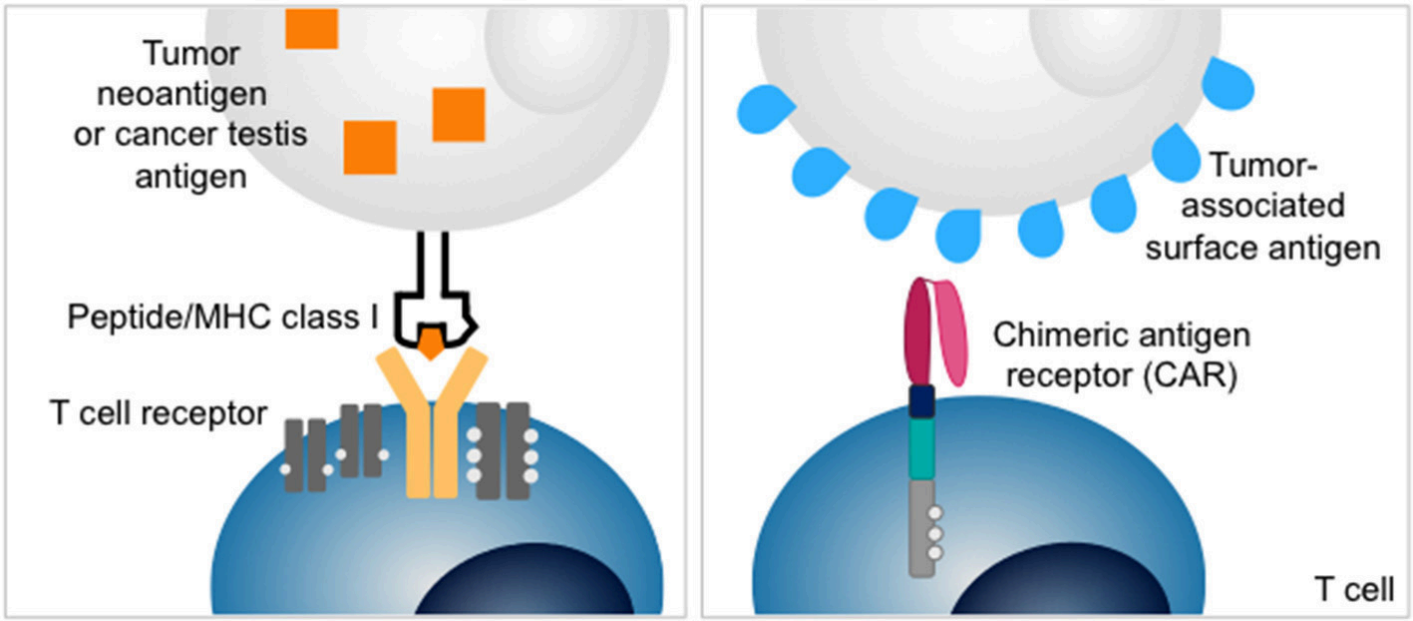
Structures of T cell receptor **(left)** and chimeric antigen receptor (CAR) **(right)**. T cell receptors recognize intracellular antigens presented as peptides on MHC class I. Chimeric antigen receptors redirect T cells to surface antigens independent of MHC-restricted antigen presentation.

The potency of T cells to control solid tumor growth is illustrated by activity of immune checkpoint inhibitors in several tumors in adults (1, 2). Most childhood tumors are not optimal candidates for this type of immunotherapy, for the following reasons [reviewed in (3)]: Checkpoint inhibitors unleash antigen-specific effector responses of T cells that were reversibly tolerized by inhibitory ligand-receptor interactions. The key prerequisite for efficacy is the presence of T cells with native specificity against tumor-associated neoantigens (4). To be recognized by T cells, the antigens must be processed and presented by the major histocompatibility complex to engage native T cell receptors (Figure 1). Tumor neoantigens are created by somatic mutations in the tumor microenvironment (5). Consequently, high non-synonymous somatic mutation frequency has emerged as one of the major prerequisites for successful T cell therapy with checkpoint inhibitors (6, 7). Most childhood tumors have very low mutational burdens, and the individual recurrent mutations typically found in childhood tumors and sarcomas are not coding for effective tumor rejection antigens. Consequently, the microenvironment of typical pediatric tumors generally is devoid of antigen-specific T cells that could potentially be reactivated by immune checkpoint inhibition (8, 9). In fact, the clinical results of early-phase trials of checkpoint inhibitors in childhood cancers are discouraging (10, 11).

An alternative strategy to exploit T cells for cancer therapy is adoptive transfer of T cells that recognize tumor-associated surface antigens by genetic modification with chimeric antigen receptors (CARs) (12). CARs link the single-chain antigen-binding domains of a monoclonal antibody to activation domains of costimulatory receptors and the T cell receptor ζ chain (Figure 1). This allows recognition of antigens expressed on the cell surface, independent of peptide presentation on MHC. CAR T cells directed against the B lineage antigen CD19 have been highly effective to control and eliminate B cell cancers in both children and adults (13–16). Intensive efforts are on the way to extend the promise of CAR T cell therapy to solid tumors. One difficulty is the identification of adequate target antigens. Effective CAR T cell therapy requires the presence of a target antigen selectively expressed on the cell surface of tumor cells but not by essential normal tissues. Examples for protein antigens targeted by CARs in advanced preclinical or early clinical development are IL13Rα (17), HER2 (18), EGFRvIII (19), CEA (20), mesothelin (21), EphA2 (22). Overall, the range of adequate antigens beyond B lineage markers is limited.

**Figure 2:**
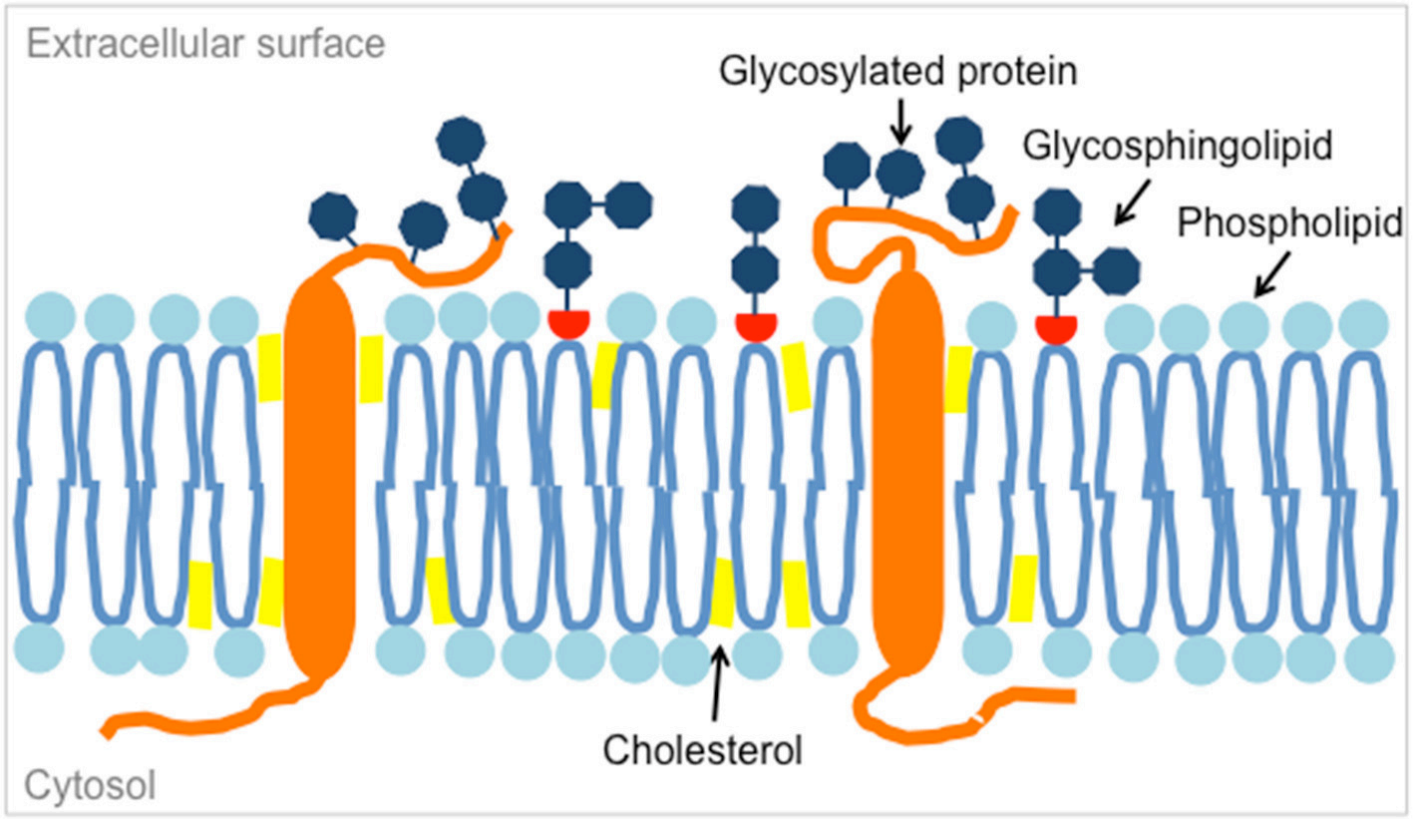
Glycosphingolipids are located in microdomains of the lipid bilayer of the plasma membrane, along with membrane phospholipids, cholesterol and transmembrane proteins such as glycosylated proteins.

Standard high-throughput screening tools used to identify novel cancer-associated antigens, including antigens enriched or preferentially expressed on the tumor cell membrane, analyse gene transcripts and proteins (23). The approaches do not cover the full spectrum of potential CAR targets. Unlike T cell receptor targets, antigens recognized by CARs include non-protein targets (Figure 2). These can be gangliosides (glycolipids/glycosphingolipids) or abnormally-glycosylated normal proteins (glycoforms) contributing to the outer glycan layer of the cell surface. Gangliosides are a subclass of glycosphingolipids characterized by the presence of one or more sialic acids. Aberrant glycosylation can include sialylation, fucosylation, *O*-glycan truncation, and *N*- and *O*-linked glycan branching. Both aberrant expression of carbohydrates and acquisition of aberrant glycosylation profiles can accompany malignant transformation. They can be unique to the malignant cell, or be restricted to immature cells but not adult somatic tissues (cancer-testis antigens), and they can contribute to tumor growth and metastasis and to immune escape. Due to their selective expression and biological role in the malignant behavior of the tumor cells, non-protein targets may be attractive target antigens. Expressed in the outer leaflet of the plasma membrane on the cell surface, they are amenable to antibody-based immunotherapeutic strategies, including antibodies (24, 25), immunotoxins, -cytokines or radioconjugates (26, 27) and more recently CAR-engineered T cells.

The first to show that CARs can redirect T cells to carbohydrates and thereby extend application of targeted T cell therapy to antigens not naturally recognized by T cells was Zelig Eshhar's group (28). They generated a CAR against the difucosylated carbohydrate Lewis-Y and found that it can effectively induce T cell lysis of antigen-expressing tumor cells despite low affinity. Lewis-Y can be coupled to various proteins and lipids, including some tumor-associated antigens. It is expressed at high levels on many types of epithelial-derived cancers as well as a proportion of cells in acute myeloid leukemia (AML) and in multiple myeloma. By contrast, it is completely absent from childhood solid tumors and other cancers of neuroectodermal or mesodermal origin (29). The first carbohydrate used as a CAR target in a pediatric cancer was the ganglioside antigen G_D2_ (30).

This review summarizes knowledge on the current most promising carbohydrate antigens for CAR T cell therapy in pediatric cancers, including G_D2_ and another ganglioside, SSEA-4, as well as aberrant glycosylation motifs.

### Glycolipid targets: gangliosides

**Figure 3:**
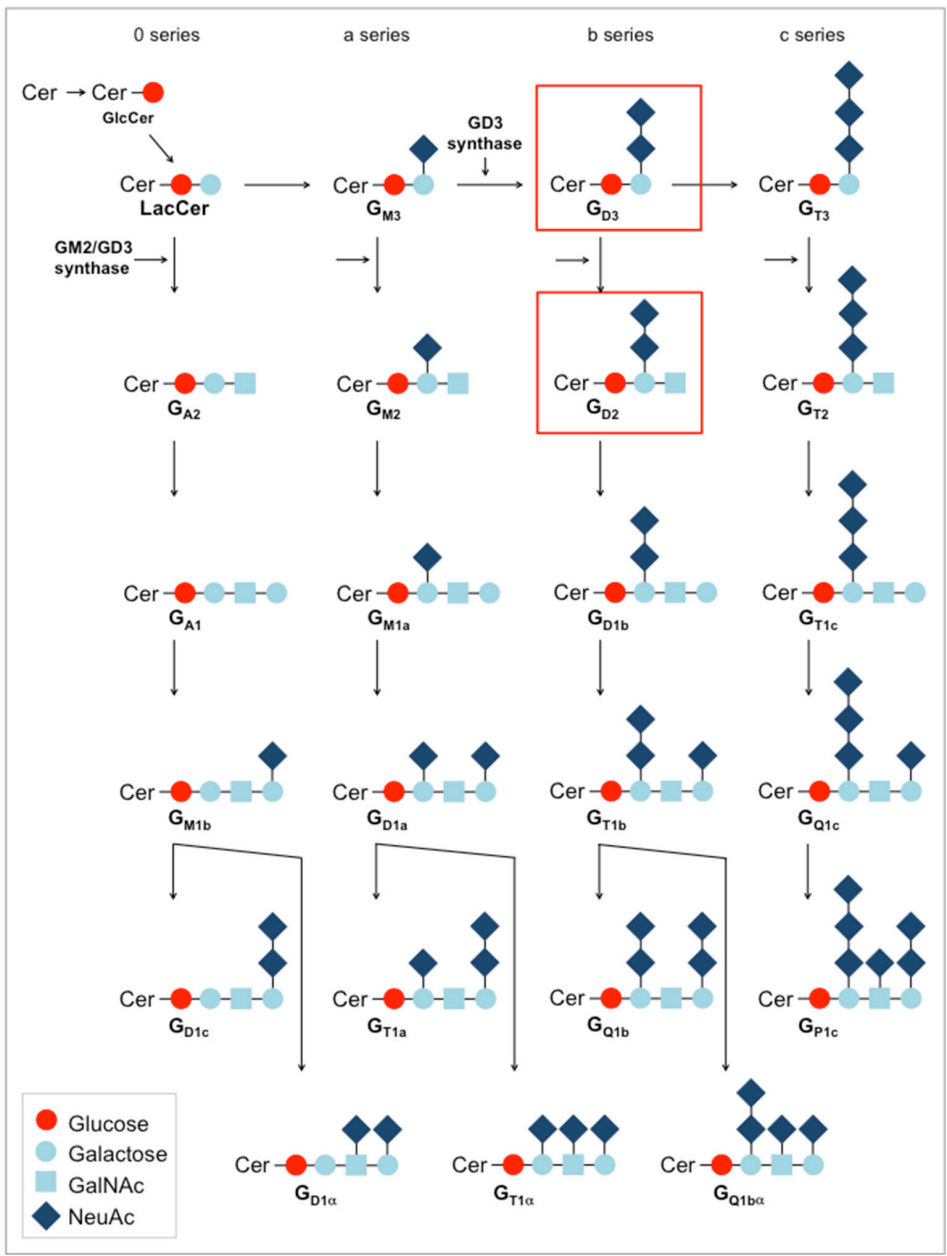
Biosynthesis pathways for gangliosides.

Gangliosides were among the first antigens exploited as tumor targets for CARs. Gangliosides are complex mammalian glycolipids. They are expressed in all vertebrates and are identical in humans and in mice and other non-human species. Located in specific microdomains of the outer leaflet of the plasma membrane, they interact with membrane phospholipids and transmembrane receptors (Figure 2). As so-called glycosynapses, carbohydrate-containing microdomains are involved in cell adhesion and subsequent signal transduction. Gangliosides have been attributed various functions in cell-cell adhesion, viability, proliferation and in the modulation of cell signaling pathways (31). Ganglioside biosynthesis is performed in the Golgi apparatus by the stepwise addition of monosaccharides to ceramide by specific glycosyltransferases along complex synthesis pathways (Figure 3). As a consequence of the stepwise synthesis pathways from its precursors, individual gangliosides are often expressed in the context of their up- and downstream epitope neighbors (32).

Individual gangliosides are expressed in a tissue-specific manner, with abundant expression on immature neural, sympathoadrenergic and mesenchymal cells during embryogenesis (33, 34, 35, 36). While the predominant gangliosides in early embryonic brains are simple gangliosides (G_M3_, G_D3_), later developmental stages are associated with more complex gangliosides with two or more sialic acid residues, such as G_D2_, G_M1a_, G_D1a_, G_D1b_, and G_T1b_ (Figure 3). After birth, expression is mainly restricted to the central nervous system where complex gangliosides at low levels are found on neuronal cell bodies. The changes in the pattern of ganglioside expression during cellular differentiation suggest specific roles of individual gangliosides at different neurodevelopmental stages. Low-level expression is also found in peripheral nerves, mesenchymal stroma cells (34), and skin melanocytes (37). The pattern of expression on healthy tissues raises important safety considerations for ganglioside-targeted therapy which will be discussed in more detail. Substantial alterations in ganglioside expression have been observed in cancer cells (38).

### Ganglioside G_D2_: expression and biology in tumor cells

Corresponding to its consistent association with immature neural crest tissue, aberrant expression of G_D2_ characterizes cancers of neuroectodermal origin, such as melanoma (39) and neuroblastoma (40). G_D2_ was further found to be expressed in several additional childhood tumors, including tumors of the CNS [retinoblastoma (41), diffuse intrinsic pons glioma (42)] and mesenchymal malignancies of the bone (Ewing sarcoma, osteosarcoma) and soft tissues (rhabdomyosarcoma, desmoplastic small round cell tumors) (43–47). Among non-melanoma cancers in adults, ganglioside G_D2_ was detected in a proportion of tumor cells in breast cancers (48) and in small cell lung cancer (49).

The mechanisms regulating expression of G_D2_ in normal human development and in cancer have not been resolved in detail. In general, expression of gangliosides during development is regulated through stage- and tissue-specific expression of ganglioside synthase genes (50). Key enzymes in G_D2_ synthesis are the glycosyltransferases G_D3_ synthase (GD3S) which synthesizes G_D3_ from G_M3_ and GM2/GD2 synthase, converting G_D3_ to G_D2_ (Figure 3). GD3S is expressed during early stages of neuronal differentiation (51). Among adult human tissues, GD3S mRNA expression is restricted to the brain. GD3S transcripts are found in G_D2_+ tumor cells of various histology (52), and surface expression of G_D2_ in breast cancer cells was found to be associated with expression of genes encoding for these enzymes, especially GD3S (53).

The activity of glycosyltransferases in normal tissues and in tumors is regulated at the transcriptional level. Studies of GD3S promoter activity in breast cancer and neuroblastoma cell lines have demonstrated a role for nuclear factor κB (NFκB) activation in transcriptional activation of the GD3S gene (54, 52). Recent studies highlight the role of epigenetic mechanisms in regulating glycosyltransferase gene expression during cellular differentiation and brain development [reviewed in (55)]. Specifically, hyperacetylation of histones was found to contribute to developmental alterations of expression of GD3S and other key enzymes of ganglioside synthesis (56, 57). Thus, epigenetic regulation of enzymes upstream of G_D2_ may be involved in the aberrant expression of G_D2_ in childhood cancers.

The biological effects of G_D2_ in both normal development and in tumors are not fully understood. G_D2_ expressed in tumor cells was found to contribute to malignant properties, including cell proliferation, invasive properties and motility (58). One potential mechanism is modulation of signal transduction along the pathways originating from G_D2_-containing glycosynapses. Indeed, examples for G_D2_-mediated activation of receptor tyrosine kinases and subsequent deregulation of critical pathways and molecules determining cell function have been reported (59, 60). Gangliosides interact with transmembrane proteins in the formation of lipid shells [reviewed in (61)]. The interaction partners of G_D2_ within the lipid microdomains and the structural relationships and downstream signaling of such interactions in normal cells and in cancer have not yet been systematically analyzed. In addition, interaction of cell surface G_D2_ with molecules in the extracellular matrix, used by neural crest cells during embryonic development to travel to distant sites, could contribute to tissue invasion and metastasis of G_D2_-positive cancer cells. In breast cancer, G_D2_ was identified as a surface marker of a subpopulation of tumor cells with specific functional properties, including self-renewal, chemoresistance, and epithelial-mesenchymal transition (EMT) (53). Inhibition of G_D2_ biosynthesis by genetic knockdown of GD3S in breast cancer cells hampered mammosphere formation, tumor initiation, and cell motility, as well as EMT and metastasis (53, 62). Besides cell-intrinsic functions, G_D2_ can contribute to immune invasion by the activation and support of immunosuppressive myeloid bystander cells in the tumor microenvironment (63). The relevance of each of these mechanisms in childhood cancers with uniform or variable G_D2_ expression, such as neuroblastoma and Ewing sarcoma or osteosarcoma, respectively, has not yet been elucidated.

### Ganglioside G_D2_ as an immunotherapeutic target

Due to its restricted tissue expression, G_D2_ is a highly ranked target for immunotherapy (64). It was first evaluated as a therapeutic target in neuroblastoma, a cancer with abundant and highly consistent G_D2_ expression reminiscent of its origin from G_D2_-positive neuroectoderm (65).

Clinical development of G_D2_ targeting started with two murine monoclonal antibodies, IgG3 antibody 3F8 and IgG2a class-switched 14.G2a, in phase I clinical trials in neuroblastoma patients in the late 1980s (24, 25). Both antibodies had similar toxicity profiles with often severe side effects including pain, fever, hypersensitivity reactions and capillary leak syndrome. Several measures were taken to enhance the activity of the antibody by promoting antibody-dependent cell-mediated cytotoxicity (ADCC) and reduce toxic side effects, especially the pain syndrome, attributed to complement activation by the Fc domain (66). Chimeric and humanized antibodies were developed to replace murine by human Fc domains to enhance ADCC (67, 68). Subsequent clinical studies used anti-G_D2_ antibody to eliminate minimal residual disease after completion of intensive multimodal treatment, either alone (69), or combined with granulocyte-macrophage colony stimulating factor GM-CSF to activate granulocytes (70), or in addition with interleukin-2 to stimulate NK cells (71). In a randomized phase III trial, the combined immunotherapeutic regimen resulted in significant increases of event-free and overall survival of high-risk neuroblastoma patients at 2 years (71). The toxicities of anti-G_D2_ antibody therapy have remained significant but are manageable by optimized supportive care (72). Although the contribution of anti-G_D2_ antibody therapy to the long-term outcomes after high-risk neuroblastoma treatment has not ultimately been proven, it is now considered a standard component of state-of-the-art treatment regimens (73). Importantly, antigen escape variants by downregulation or loss of G_D2_ expression in response to G_D2_-specific targeting have been rare (71, 74, 75), supporting the value of this antigen in neuroblastoma.

Redirecting T cells to G_D2_ exploits the trafficking qualities and potent effector functions of T cells and thus could be a more effective strategy to target G_D2_-expressing tumor cells. The first CAR generated against G_D2_ was derived from the single-chain Fv (scFv) domains of monoclonal antibody 14.G2a (76), linked to the T cell receptor ζ chain (30). 14.G2a-redirected CAR T cells specifically and effectively lysed G_D2_-positive neuroblastoma cells *in vitro*. Since soluble G_D2_ is present at high concentrations in serum of advanced-stage neuroblastoma patients, competitive binding and blockade of G_D2_-directed CAR T cell activity had to be excluded. Soluble G_D2_ did not impede tumor cell lysis by 14.G2a-CAR T cells i*n vitro* (30). Lack of a competing effect of shed antigen on the antitumor activity of CAR-redirected T cells had previously been shown for another carbohydrate target, Lewis-Y (77), and also for protein antigens shed into the blood stream by tumor cells such as CEA (78).

In an early clinical trial, treatment of neuroblastoma patients with autologous virus-specific T cells expressing the anti-G_D2_ CAR was safe, with some evidence of activity (79–81). Further phase I clinical studies have used signal-enhanced CARs and refined treatment regimens (82, 83). The presence of G_D2_ at low levels on neuronal cell bodies has caused significant safety concerns for the clinical use of G_D2_-specific CAR T cells. Whereas, the brain is protected from intravenous infusions of G_D2_ antibody by the blood-brain barrier, CAR T cells effectively penetrate into the CNS. Neither of the clinical trials performed so far has shown any evidence of neurotoxic side effects or pain (79, 80, 83), and this includes a recent trial demonstrating impressive clinical responses associated with tumor lysis syndrome and cytokine release (83). Thus, the lack of significant on-target off-tumor toxicities in the central and peripheral nervous system cannot be attributed to lack of activity. As the 14.G2a-derived G_D2_-specific CAR was designed to contain the isolated scFv fragment without any immunoglobulin heavy chain components to bridge it from the plasma membrane, the lack of any pain side effects is consistent with the hypothesis that activation of complement by the Fc domain of anti-G_D2_ antibodies is the responsible mechanism for this side effect (66). In contrast to clinical trials, neurotoxicity was reported in a mouse model following treatment with T cells expressing an affinity-enhanced 14.G2a-based CAR (84). Clinical signs of encephalopathy in this model were associated with T cell infiltration in brain regions with low-level G_D2_ expression. Whether the clinical picture indeed represented on-target cross-reactivities with murine brain or cytokine-mediated off-target toxicities, as well-known from the use of CD19-specific CAR T cells, remains controversial (85). In further xenograft models, potent antitumor activity of G_D2_-specific CAR T cells was not associated with neurotoxicities (86, 45). This encouraged the development of this therapy even for G_D2_-positive CNS tumors (42). In preclinical studies, G_D2_-specific CAR T cells were highly active against G_D2_-positive diffuse intrinsic pons glioma xenografts in the CNS. Neurological symptoms occurring in some of the animals were associated with tumor swelling by pseudoprogression, without histological damage of brain tissue. As long as safety concerns remain, the use of NK cells rather than long-lived T cells could be a safer alternative to targeting G_D2_-positive tumors (44).

Preclinical data support the value of G_D2_-specific CARs also for immunotherapy of G_D2_-expressing sarcomas, such as Ewing sarcomas (44, 87, 88) and osteosarcomas (45). In contrast to neuroblastomas, only a proportion of these tumors express significant levels of G_D2_, and antigen expression is often heterogeneous (45, 87). Thus, G_D2_-specific immunotherapy in childhood sarcomas will have to be combined with strategies eliminating also G_D2_low and G_D2_neg tumor cell subpopulations and preventing antigen-negative immune escape.

A highly related variant of G_D2_ is O-acetyl-G_D2_ (OAcGD2), characterized by a 9-O-acetyl modification on the terminal sialic acid of G_D2_. While tumors that express G_D2_ were generally found to also express the O-acetylated variant, human peripheral nerve fibers do not express OAcGD2 (89). Consequently, antibodies targeting this variant were developed to avoid the allodynic properties of G_D2_ (90). Whether selective recognition of the O-acetylated variant of G_D2_ is preferable for G_D2_-specific CARs is not clear.

### Ganglioside G_D3_: an alternative or complementary immune target?

G_D3_ is a b-series ganglioside containing two sialic acids. It is produced from its precursor G_M3_ by the activity of GD3S. Physiologically it is highly expressed in embryonic neural stem cells (36). Its interest as a tumor antigen has mostly been in melanoma where G_D3_ is highly expressed, with no or minimal levels of G_D3_ on human normal melanocytes and restricted expression on other normal tissues to low levels on retinal pigment cells and in the CNS. G_D3_ has been investigated as a target for antibody therapy in melanoma (91, 92), and anti-G_D3_ CARs are in preclinical development (93, 94).

While neuroblastomas have only moderate expression of G_D3_ compared to G_D2_, an immunohistochemical analysis of various childhood sarcomas has demonstrated a high prevalence of G_D3_ expression especially in osteosarcomas, but also in a proportion of Ewing sarcomas and rhabdomyosarcomas (43). Moreover, malignant gliomas express high levels of G_D3_ as well as O-acetylated G_D3_ and GD3S (95, 96), and expression is associated with the degree of malignancy (96) and with neurosphere formation and clonogenic properties (97). A G_D3_-specific antibody, acting via complement-dependent cytotoxicity, was found to inhibit glioblastoma tumor growth in an *in vivo* model (97).

Thus, G_D3_ could be a CAR target in both CNS tumors and extracranial tumors in the pediatric population, either alone or in combination with G_D2_, to broaden T cell recognition in cancers with heterogeneous expression of either of the two gangliosides. However, potential on-target/off-tumor side effects by reactivity of G_D3_-specific CAR T cells with CNS tissues, especially with the retina, will have to be studied diligently.

### Ganglioside N-Glycolyl G_M3_ (NeuGcGM3)

G_M3_ is a monosialoganglioside and the direct precursor of G_D3_ (Figure 3). Whereas, the acetylated form, N-acetyl G_M3_, is abundant in normal tissues, humans in contrast to other mammals cannot generate N-glycolylated G_M3_ (NeuGcGM3) due to a constitutional deletion in the gene encoding the enzyme which catalyzes the conversion of N-acetyl to N-glycolyl sialic acid (98). Expression of NeuGcGM3 was observed in human cancers and explained by expression of the sialic acid transporter under hypoxic conditions, resulting in incorporation of non-human sialic acid from dietary supplies (99). In fact, natural xeno-autoantibodies against NeuGcGM3 were found in human serum and correlated with the presence of cancers (100). Among pediatric tumors, neuroblastomas, Wilms tumors, Ewing sarcomas and retinoblastomas were reported to express NeuGcGM3 (101) whereas various normal tissues were negative [summarized in (102)]. An anti-idiotype vaccine, racotumumab, was able to induce antibody responses to the target in a phase I clinical trial in refractory childhood cancers, without any evidence for off-tumor toxicities (103). Direct antibody or CAR targeting of this antigen has not yet reached clinical translation. Again, although immunoreactivity of the anti-NeuGcGM3 antibody has so far been restricted to tumor tissues, interactions with healthy tissues, with the potential to cause limiting toxicities, cannot be excluded.

### Stage-specific embryonic antigen-4 (SSEA-4): a marker for embryonic stem cells

**Figure 4:**
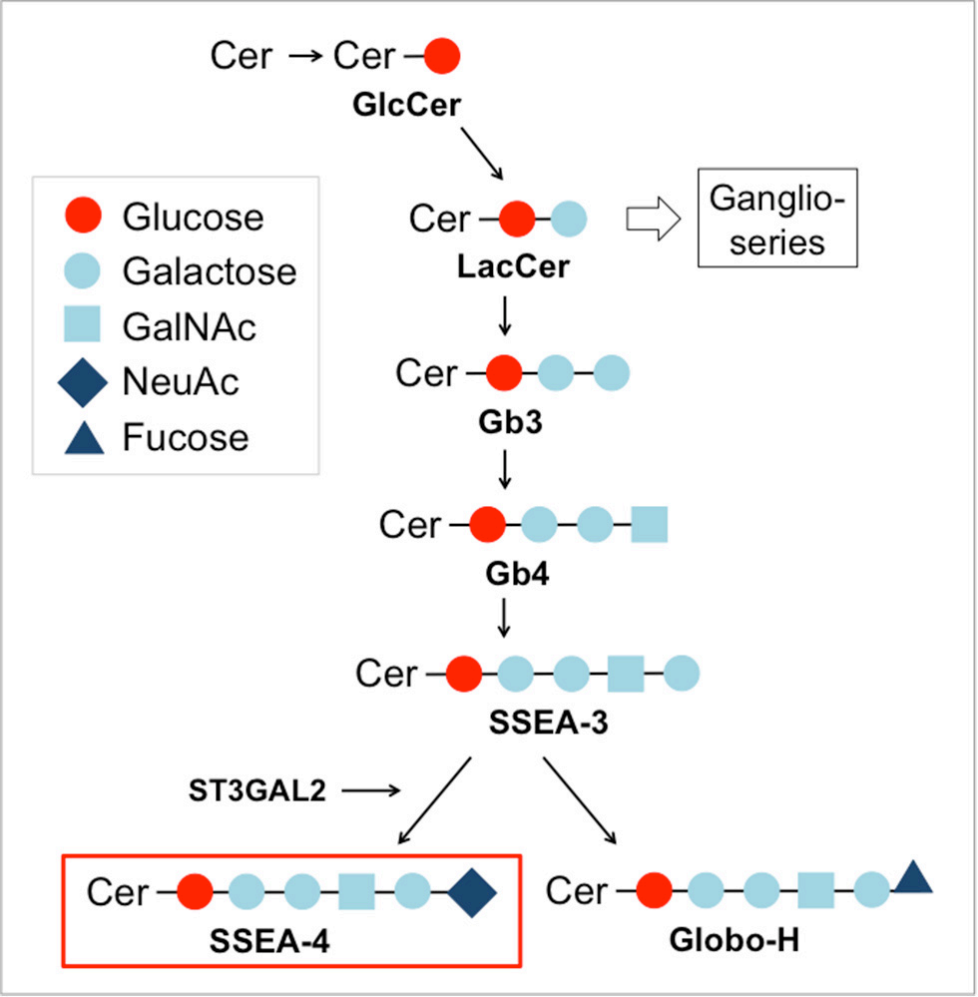
Biosynthesis pathway for globo-series gangliosides.

SSEA-4 is a globo-series ganglioside synthesized from SSEA-3 by the enzyme ST3 beta-galactoside alpha-2,3-sialyltransferase 2 (ST3GAL2) (104) (Figure 4). As other glycosphingolipids, SSEA-4 is a component of glycosynapses of the plasma membrane (Figure 2). Due to its highly restricted expression in pluripotent human embryonic stem cells, SSEA-4 conceptually is an attractive target for CAR T cell therapy. During human preimplantation development, SSEA-4 is first observed on the pluripotent cells of the inner cell mass and lost upon differentiation (105). After birth, human germ stem cells in the testis and ovary (106, 107) as well as mesenchymal stem cells (108) express SSEA-4. Its biological function has not yet been resolved in detail. In human tumors, SSEA-4 was first identified in a teratocarcinoma cell line (109). It was further found to be overexpressed in osteosarcoma (110), prostate cancer (111), breast cancer (112), and glioblastoma (113).

Several studies suggest that SSEA-4 expression in cancer marks subpopulations with specific biological properties within the tumor bulk. Among osteosarcoma cells, SSEA-4-positive subpopulations, but not SSEA-4-negative tumor cells, reliably established xenografts in mice, and in patients with this cancer, the frequency of SSEA-4 expressing tumor cells was inversely correlated with overall survival (110). Moreover, tumor tissue extracted after chemotherapy showed a higher number of SSEA-4-positive tumor cells, suggesting selection by chemotherapy, and/or an upregulation of the antigen by cytotoxic agents (110). In prostate cancer, SSEA-4 expressing cell subpopulations were highly tumorigenic, had higher cellular adhesion and a migratory phenotype, indicating a role of SSEA-4 in cancer invasion (111). SSEA-4 in breast cancer cells was found to be highly expressed in a subpopulation of chemotherapy-resistant tumor cells, and expression of enzyme ST3GAL2 was a predictive marker for poor outcome (112). In astrocytomas, SSEA-4 expression was associated with higher tumor grades (113).

Selective presence in cancer cells and association with self-renewing and migratory properties makes SSEA-4 a candidate target for antibody-based immunotherapies. In preclinical experiments, the SSEA-4 specific monoclonal antibody MC 813-70 was indeed active to inhibit the growth of glioblastomas in nude mice (113). Apparent toxicities were not described in this model (113). By contrast, SSEA-4-specific CAR T cells were found to cause major alterations in the composition of the hematopoietic compartment in a preclinical mouse model, suggestive of on-target/off-tumor toxicity (114). Whether SSEA-4 expression in humans after birth is sufficiently restricted to cancer cells to allow for safe targeting of cancer with CAR-engineered T cells remains an open question.

### Carbohydrate modifications of cell surface proteins

Besides membrane glycolipids, aberrant glycosylation of cell surface proteins in cancers leads to expression of distinct glycoproteins which could allow for selective CAR targeting. Cancer-associated cell surface glycosylation was shown to be directly involved in malignant transformation and metastasis [reviewed in (115)], supporting the potential of glyco-epitopes as immune targets. The most prevalent glyco-epitopes in carcinomas are Tn (GalNAcα1-Ser/Thr) and sialyl-Tn (STn, NeuAcα2,6GalNAc-Ser/Thr). Tn and STn are present on many glycoproteins expressed in epithelial cancers, including MUC1 (116, 117). MUC1 carring such variations was associated with poor prognosis, reduced chemosensitivity and with immune-inhibitory properties in breast cancer (116).

The first CARs against MUC1 were directed against the protein core. Their tumor selectivity was mediated by MUC1 hypoglycosylation which enabled the scFv to access the protein (118). More recently, CARs were directed to specific truncated O-glycopeptide epitopes not expressed on normal tissues and shown to differentiate between wild-type and Tn-glycoforms of MUC1 (117). CAR T cells against Tn-MUC1 effectively controlled tumor growth in murine xenograft models. Thus, aberrant glycosylation can turn a self-antigen into a neoantigen which can serve as target for tumor rejection by CAR T cells.

Proteins that recognize glycans, so-called lectins, could be alternative recognition domains for CARs targeting aberrant glycosylation. In a recent report, lectins were identified that specifically bind to a fucosylated glycan epitope on the surface of pancreatic cancer cell lines (119).

In pediatric cancers, oncofetal chondrosulfate (ofCS) modifications of glycosaminoglycans attached to proteoglycans on the cell surface have potential to serve as CAR targets (120). Chondroitin sulfate chains normally restricted to the placenta are found on many cancer-associated proteoglycans, including CSPG4, syndecan-1,−4, CD44, and glypican-1, with widespread expression also in pediatric tumors (120). Placental ofCS is detected by malaria protein VAR2CSA, suggesting a role in pregnancy-associated malaria (120). Studies using VAR2CSA as a model ligand for ofCS demonstrated a key role of this modification in the malignant phenotype, especially in tumor cell motility and metastatic potential (121). The ligand was further found to be able to isolate circulating tumor cells from peripheral blood (122). Thus, ofCS could be both a marker and a therapeutic target of the highly metastatic subpopulation of tumor cells across various types of cancer, including childhood solid tumors. A concern is the variability of protein glycosylation, which despite the biological function of the oncofetal modification could allow easy escape from immune targeting. Moreover, lack of expression on normal tissues besides placenta will have to be unequivocally demonstrated.

### Outlook

The feasibility of redirecting T cells against carbohydrate antigens which they do not normally recognize using CARs has been well demonstrated in both preclinical and clinical studies. Still, among the large numbers of CAR targets currently explored, relatively few are non-protein antigens. Carbohydrate antigens may be under-studied for technical reasons. Immunohistochemistry detection of carbohydrates is often limited to cryopreserved material not generally available from biopsies of pediatric tumors. Gangliosides and other glycolipids do not appear in gene and protein expression analyses, and genes encoding for the enzymes necessary for ganglioside synthesis are not useful surrogates to predict expression of specific gangliosides since these are products of numerous glycoslytransferases. A major challenge to the identification of cancer-associated glycosylation variants is their diversity and heterogeneity. Known carbohydrate targets in cancer were identified by their interaction with monoclonal anti-tumor antibodies followed by analysis of the chemical structure of the antibody target. Novel mass spectrometry-based technologies in modern glycomics now allow systematic and high-throughput comparative analysis of the patterns of gangliosides and oligosaccharides in tissues, followed by purification and structural elucidation (123–125). Glycomic screening of the cancer cell surface in pediatric cancers could be a useful next step to identify novel carbohydrate targets for CAR gene-modified T cells (126).

Many questions with regard to the tissue specificity and biology of non-protein antigens are still unanswered. Many gangliosides are physiologically expressed in a stage- and tissue-specific manner in human embryogenesis and could be present on neuronal, mesenchymal or hematopoietic stem cells after birth, raising important safety concerns. The mechanisms regulating tissue-specific expression of individual gangliosides are often unknown but highly relevant to better understand their biological role. This knowledge may also serve to find interventions that upregulate presence of the antigen on cancer cells with heterogeneous antigen expression, a major limitation for effective tumor targeting by CAR T cells.

While it is unlikely that carbohydrate targets with restricted expression on tumor cells will be found in the majority of childhood cancers, novel T cell engineering strategies may allow safe targeting of antigens despite low level expression in normal tissues. One approach is combinatorial antigen recognition, resulting in full T cell activation responses only in the presence of two or more target antigens. For this purpose, T cells can be cotransduced with a CAR that provides suboptimal activation in response to antigen, along with a chimeric costimulatory receptor that recognizes a second antigen (127). Alternatively, modular synthetic receptors were designed on the basis of the capacity of Notch receptors to perform transcriptional switches (128). Engagement of a first CAR by its target antigen releases an intracellular synthetic transcription factor that selectively induces gene expression of a second CAR. In a mouse model, this resulted in selective clearance of tumors coexpressing both antigens. An alternative strategy to protect off-target tissues against CAR T cells is cotransduction with an activating CAR and a CAR that delivers inhibitory signaling in response to a second antigen expressed exclusively on normal cells (129).

Moreover, control systems have been developed that allow to remove T cells on demand in cases of on- or off-target toxicities. Such suicide switches rely on genes that render the cells sensitive to prodrugs (130, 131) or proapoptotic genes activated by dimerization(132), or on surface markers for antibody-mediated depletion, e.g., truncated epithelial growth factor receptor (tEGFR) (133) or rituximab(134). An even safer solution could be “on-switch” CARs. Separation of intracellular or costimulatory signals from extracellular antigen recognition in inducible gene expression systems allows to turn on the respective signaling pathway by small molecules on demand (135, 136). In the view of the rapidly expanding toolbox for tuning and controlling the *in vivo* activity and persistence of CAR T cells, carbohydrate antigens as well as protein antigens with and without carbohydrate modifications, despite low-level coexpression on healthy tissues, deserve investigation as potential targets for future generations of CARs.

## Author contributions

All authors listed have made a substantial, direct and intellectual contribution to the work, and approved it for publication.

### Conflict of interest statement

The authors declare that the research was conducted in the absence of any commercial or financial relationships that could be construed as a potential conflict of interest.
